# Prevalence of International Medical Graduates From Muslim-Majority Nations in the US Physician Workforce From 2009 to 2019

**DOI:** 10.1001/jamanetworkopen.2020.9418

**Published:** 2020-07-06

**Authors:** John R. Boulet, Robbert J. Duvivier, William W. Pinsky

**Affiliations:** 1 Educational Commission for Foreign Medical Graduates, Philadelphia, Pennsylvania; 2 Foundation for Advancement of International Medical Education and Research, Philadelphia, Pennsylvania; 3 Parnassia Psychiatric Institute, The Hague, the Netherlands

## Abstract

**Importance:**

Historically, the US physician workforce has included a large number of international medical graduates (IMGs). Recent US immigration policies may affect the inflow of IMGs, particularly those who are citizens of Muslim-majority nations.

**Objectives:**

To provide an overview of the characteristics of IMGs from Muslim-majority nations, including their contributions to the US physician workforce, and to describe trends in the number of applications for certification to the Educational Commission for Foreign Medical Graduates between 2019 and 2018, both overall and for citizens of Muslim-majority nations.

**Design, Setting, and Participants:**

This cross-sectional study, which included 1 065 606 US physicians listed in the 2019 American Medical Association Physician Masterfile and 156 017 applicants to the Educational Commission for Foreign Medical Graduates certification process between 2009 and 2018, used a repeated cross-sectional study design to review the available data, including country of medical school attended, citizenship when entering medical school, and career information, such as present employment, specialty, and type of practice.

**Exposures:**

Country of citizenship when entering medical school.

**Main Outcomes and Measures:**

Physician counts and demographic information from the 2019 American Medical Association Physician Masterfile and applicant data from the Educational Commission for Foreign Medical Graduates from 2009 to 2018.

**Results:**

Of 1 065 606 physicians in the American Medical Association Physician Masterfile, 263 029 (24.7%) were IMGs, of whom 48 354 were citizens of Muslim-majority countries at time of entry to medical school, representing 18.4% of all IMGs. Overall, 1 in 22 physicians in the US was an IMG from a Muslim-majority nation, representing 4.5% of the total US physician workforce. More than half of IMGs from Muslim-majority nations (24 491 [50.6%]) come from 3 countries: Pakistan (14 352 [29.7%]), Iran (5288 [10.9%]), and Egypt (4851 [10.0%]). The most prevalent specialties include internal medicine (10 934 [23.6%]), family medicine (3430 [7.5%]), pediatrics (2767 [5.9%]), and psychiatry (2251 [4.8%]), with 18 229 (38.1%) practicing in primary care specialties. The number of applicants for Educational Commission for Foreign Medical Graduates certification from Muslim-majority countries increased from 2009 (3227 applicants) to 2015 (4244 applicants), then decreased by 2.1% in 2016 to 4254 applicants, 4.3% in 2017 to 4073 applicants, and 11.5% in 2018 to 3604 applicants. Much of this decrease could be attributed to fewer citizens from Pakistan (1042 applicants in 2015 to 919 applicants in 2018), Egypt (493 applicants in 2015 to 309 applicants in 2018), Iran (281 applicants in 2015 to 182 applicants in 2018), and Saudi Arabia (337 applicants in 2015 to 163 applicants in 2018) applying for certification.

**Conclusions and Relevance:**

Based on the findings of this study, the number of ECFMG applicants from Muslim-majority countries decreased from 2015 to 2018. The US physician workforce will continue to rely on IMGs for some time to come. To the extent that citizens from some countries no longer seek residency positions in the US, gaps in the physician workforce could widen.

## Key Points

**Question:**

What is the prevalence of international medical graduates from Muslim-majority nations in the US physician workforce, and what are the trends in this group’s contribution to the labor force?

**Findings:**

In this cross-sectional study, citizens from Muslim-majority nations made up 4.5% of the US physician workforce in 2019. Applications to the Educational Commission for Foreign Medical Graduates increased from 2009 to 2015 and decreased from 2016 to 2018.

**Meaning:**

The findings of this study suggest that the contribution of international medical graduates from Muslim-majority nations is likely to decrease, which may exacerbate gaps in the US physician workforce.

## Teaser

This cross-sectional study provides an overview of the characteristics of international medical graduates from Muslim-majority nations, including their contributions to the US physician workforce, and describes trends in the number of applications for certification to the Educational Commission for Foreign Medical Graduates between 2019 and 2018, both overall and for citizens of Muslim-majority nations.

## Introduction

International medical graduates (IMGs) represent approximately one-quarter of practicing physicians in the US, including residents.^1^ The countries of origin of these IMGs have varied over time, but historically, most received their medical degrees from schools located in the Caribbean, India, Mexico, Pakistan, and the Philippines.^2,3^

International medical graduates who wish to enter graduate medical education (GME) programs in the US must first be certified by the Educational Commission for Foreign Medical Graduates (ECFMG). Current ECFMG certification requirements include verification of the medical school diploma and passing performance on the US Medical Licensing Examination (USMLE) Step 1, Step 2 Clinical Knowledge, and Step 2 Clinical Skills. To obtain an unrestricted license to practice medicine in any US jurisdiction, IMGs must complete 2, and often 3, years of training in an accredited GME program. While there are many challenges associated with physician migration to the US, the more than 250 000 IMGs presently in the US attest to their motivation and desire for quality education and practice opportunities.

A shifting political and health care landscape has affected both the pipeline of IMGs (those seeking residency training in the US) as well as the practicing IMG population. The underlying reasons for these changes are multifaceted. Demand for physicians has grown faster than supply. Population growth and aging are the primary drivers for increasing demand. A 2015 report by the American Association for Medical Colleges predicted a shortage of 42 600 to 121 300 physicians by 2030.^4^ Competition for GME positions has also increased. In response to projected workforce shortfalls, the number of domestic medical schools has increased.^5^ During the last decade, 25 new schools have received accreditation from the Liaison Committee on Medical Education. In the past 10 years, student matriculation among MD-granting institutions increased by 19.9%, from 18 036 in 2008 to 21 622 in 2018.^6^ The number of osteopathic graduates was 3588 in 2008 to 2009 and 6416 in 2017 to 2018, representing a 78.8% increase.^7^ Unfortunately, the growth in the number of GME positions has not kept pace, in part because of a federal cap on funding for residency training.^8,9,10^ As a result, and pending the full implementation of a single accreditation pathway for GME programs,^11^ IMGs may encounter even more competition for GME positions.

Geopolitical forces can also affect the migration of professionals among countries. Instability because of political upheaval or armed conflict has determined outflow, whereas immigration policies, certification and licensure requirements, and financial resources have regulated inflow. For example, changes to the certification requirements have led to considerable variations in the number of medical students and graduates seeking ECFMG certification.^2^ In 1992, application rates increased before the introduction of the USMLE, suggesting that IMGs were trying to avoid this new requirement. In 1999, application rates decreased in response to the addition of the Clinical Skills Assessment as an ECFMG certification requirement in 1998. Certification and licensure requirements, including the costs of the examinations, likely have some effect on physician immigration, especially from low-resource countries.^2^

Recent US policies might also affect the inflow of IMGs by restricting travel to the country for citizens from specific nations, in particular, President Trump’s executive order 13780, “Protecting the Nation from Foreign Terrorist Entry into the United States.”^12^ It is referred to as the Muslim ban by critics^13,14,15^ because it targets Muslim-majority countries. The executive order has been redrafted, and certain provisions have been overturned in the courts. While the direct effect of such policies on IMGs is unknown,^16,17,18^ even a perceived immigration ban could affect who chooses to complete the requirements for ECFMG certification and compete for GME positions in the US.^19^ Immigration issues could also affect program directors’ National Residency Matching Program ranking decisions because they risk their positions remaining unfilled if a successful IMG candidate who is not a US citizen fails to obtain the required visa for working in the US.

The primary purpose of this study was to provide an overview of the characteristics of IMGs from Muslim-majority nations, including their contributions to the US physician workforce. The secondary purpose was to describe ECFMG application trends from 2009 to 2018, comparing overall trends with those for citizens of Muslim-majority nations. Given that many ECFMG applicants will eventually meet the certification requirements and most certificate holders eventually obtain a GME position in the US,^2^ it is important to try to understand how the future composition of the physician workforce could change. We note that most reports on IMG migration are based on country of medical school,^20,21^ which ignores the reality that medical students are mobile in pursuing their education.^22^ Because the executive order, like all immigration legislation, applies to citizenship, country of medical school training does not accurately capture the population of interest. Therefore, we defined our analysis cohorts by citizenship at time of entry to medical school, not country of medical school training.

## Methods

This cross-sectional study used information from the 2019 American Medical Association Physician Masterfile^23^ (AMA Masterfile) combined with data from ECFMG. To better understand the contribution of citizens from Muslim-majority nations to the US workforce, we provide a descriptive profile of their characteristics based on population data from the 2019 AMA Masterfile. To explore potential changes to the composition of the IMG workforce in the US, we analyzed trends in ECFMG applications between 2009 and 2018. Part of the process of data merging consisted of using a unique identifier to match individuals across both data sets. The final data set used for analysis was fully anonymized, and data cannot be traced back to individual physicians. Inclusion in either data set was done with consent of the individuals involved, in the case of the AMA as part of their member application process and, for the ECFMG, as part of the application for certification. In this application, the candidate must agree to allow their data to be used for research. If they do not agree, their record is not included in any analysis. Because the data are publicly available and all participants agreed that their anonymized data could be used for research purposes, the study was deemed exempt by the Internal Research and Data Review Committee of the ECFMG. The Strengthening the Reporting of Observational Studies in Epidemiology (STROBE) reporting guideline was followed in conducting this investigation.^24^

The AMA Masterfile contains data on all physicians in the US who have met the requirements to practice. Graduates from US medical schools are listed when they enter any medical school accredited by the Liaison Committee on Medical Education. International medical graduates are included when they enter a residency training program accredited by the Accreditation Council for Graduate Medical Education. Additional information is obtained through primary sources and physician surveys. Data include demographic characteristics, such as age and country of birth, and information on career markers, such as year of graduation, specialty, type of practice, and present employment.

The ECMFG data was linked to the AMA Masterfile using unique identifiers. For the purpose of this study, information from ECFMG included country of citizenship at the time of entry into medical school, country of medical school training, and medical school attended.

### Study Population

#### IMGs From Muslim-Majority Nations

Muslim-majority nations are those where at least 50% of the population is Muslim. Based on a number of sources,^25,26,27,28^ Muslim-majority nations are Afghanistan, Albania, Algeria, Azerbaijan, Bahrain, Bangladesh, Bosnia and Herzegovina, Burkina Faso, Djibouti, Egypt, Guinea, Guinea-Bissau, Indonesia, Iran, Iraq, Jordan, Kazakhstan, Kosovo, Kuwait, Kyrgyzstan, Lebanon, Libya, Malaysia, Mali, Mauritania, Morocco, Niger, Nigeria, Oman, Palestine, Pakistan, Qatar, Saudi Arabia, Senegal, Somalia, Sudan, Syria, Tajikistan, Tunisia, Turkey, Turkmenistan, United Arab Emirates, Uzbekistan, and Yemen. For this study, citizenship at entry to medical school was used to delimit IMGs who were citizens of Muslim-majority nations and IMGs who were US citizens.

#### Active Physicians

The AMA Masterfile contains information on physicians practicing in the US, including type of practice and major professional activity. The AMA data also contain information on practice specialty. We used data from physicians who were active, eliminating all physicians whose major professional activity was inactive, who were presumed dead, and who were older than 90 years. More specifically, the active category included physicians whose major professional activity was classified as full-time hospital staff, resident, research, office-based practice, administration, medical teaching, student, other, locum tenens, semiretired, and not classified.

#### Applicants

For this study, an ECFMG applicant was defined as an individual who registered and sat for 1 of the examinations required for ECFMG certification. Sitting for an examination required for ECFMG certification is a reasonable indicator of intent to secure GME training in the US. The examination requirements for certification are described elsewhere.^29^

### Statistical Analysis

Descriptive statistics were used to characterize the different groups of IMGs and to quantify their contribution to the US physician workforce. Because the population of active physicians in the US and ECFMG applicants were available for analysis, no inferential statistics were calculated. Data analysis was conducted with SAS version 9 (SAS Institute).

## Results

### Citizens of Muslim-Majority Nations in the US Physician Workforce

Based on the 2019 AMA Masterfile, there were 1 065 606 physicians in the US whose major professional activity is not inactive. Nearly one-quarter (263 029 [24.7%]) of these physicians were IMGs. Within the IMG cohort, 48 354 (18.4%) were citizens of Muslim-majority countries at time of entry to medical school. IMGs from Muslim-majority nations represented 4.5% of the total physician workforce.

Table 1 provides the country of citizenship for the top 20 Muslim-majority countries. More than half of IMGs from Muslim-majority nations were citizens of 3 countries: Pakistan (14 352 [29.7%]), Iran (5288 [10.9%]), and Egypt (4851 [10.0%]). Based on country of medical school training, 4077 IMGs from Muslim-majority countries (8.4%) did not attend medical school in Muslim-majority countries. Many of these individuals attended medical school in Dominica (370 [9.1%]), Grenada (339 [8.3%]), and the Dominican Republic (322 [7.9%]).

**Table 1.** Country of Citizenship at Entry to Medical School for International Medical Graduates From Muslim-Majority Countries

Country of citizenship	No. (%) (n = 48 354)
Pakistan	14 352 (29.68)
Iran	5288 (10.94)
Egypt	4851 (10.03)
Syria	4580 (9.47)
Nigeria	4301 (8.89)
Lebanon	3561 (7.36)
Jordan	2408 (4.98)
Iraq	1614 (3.34)
Bangladesh	1556 (3.22)
Turkey	1423 (2.94)
Saudi Arabia	894 (1.85)
Sudan	644 (1.33)
Malaysia	469 (0.97)
Libya	348 (0.72)
Afghanistan	280 (0.58)
Indonesia	256 (0.53)
Palestinian territory	209 (0.43)
Albania	170 (0.35)
Morocco	145 (0.30)
Algeria	135 (0.28)
All other Muslim-majority countries	870 (1.80)

The IMGs from Muslim-majority countries worked primarily in office-based practice (29 124 [60.2%]) and as full-time hospital staff (6395 [13.2%]). There were 5731 IMG residents from Muslim-majority countries, representing 20.9% of 27 424 IMG residents. Based on type of practice, most IMGs from Muslim-majority countries were in direct patient care (35 366 [73.1%]). Their most prevalent specialties, based on self-designated practice specialty, included internal medicine (10 934 [23.4%]), family medicine (3430 [7.1%]), pediatrics (2767 [5.9%]), and psychiatry (2251 [4.8%]). Nearly 40% (18 229 [38.1%]) were practicing in primary care specialties. The top 5 practice states for IMGs, including the number and percentage of active IMGs from Muslim-majority countries, are presented in Table 2. For these states, more than 16% of active IMGs were citizens of Muslim-majority countries at entry to medical school (19 106 of 115 523 [16.5%]). More than 25% of all IMGs from Muslim-majority countries graduated from 1 of the 5 following medical schools: Dow Medical College, Pakistan (3433 [7.1%]); University of Damascus Faculty of Medicine, Syria (2897 [6.0%]); King Edward Medical University, Pakistan (2150 [4.5%]); American University of Beirut Faculty of Medicine, Lebanon (1952 [4.0%]); and Tehran University of Medical Sciences School of Medicine, Iran (1727 [3.6%]).

**Table 2.** Top 5 Practice States for IMGs

State	Active IMGs, No.	IMGs from Muslim-majority countries, No. (%)
New York	32 322	5082 (15.7)
California	28 666	5103 (17.8)
Florida	22 850	2850 (12.5)
Texas	18 777	4050 (21.6)
New Jersey	12 908	2021 (15.7)

Abbreviation: IMG, international medical graduates.

### Historical Trends for ECFMG Applicants From Muslim-Majority Countries 

Since 1958, there have been nearly 664 000 applications for ECFMG certification (when an initial examination year was available). Of these, 145 844 (21.9%) were citizens of Muslim-majority countries. The total number of applicants who were citizens of Muslim-majority countries, citizens of non–Muslim-majority countries excluding US citizens, and US citizens is provided in Table 3.

**Table 3.** Educational Commission for Foreign Medical Graduates Applicants by Citizenship Status at Entry to Medical School, 2009-2018

Application year	IMGs from Muslim-majority nations	Change (%)	IMGs from non–Muslim-majority and non-US nations	Change (%)	IMGs with US citizenship	Change (%)	Total	Change (%)
2009	3227	NA	7918	NA	3694	NA	14 839	NA
2010	3587	360 (11.2)	7861	−57 (−0.7)	3701	7 (0.2)	15 149	310 (2.1)
2011	3812	255 (6.3)	8106	245 (3.1)	4011	310 (7.3)	15 929	780 (5.1)
2012	3723	−89 (−2.3)	7507	−599 (−7.4)	4063	52 (1.3)	15 294	−635 (−4.0)
2013	4024	301 (8.1)	7691	184 (2.5)	4049	−14 (−0.3)	15 764	470 (3.1)
2014	4228	204 (5.1)	7747	56 (0.7)	4183	134 (3.2)	16 188	424 (2.7)
2015	4344	116 (2.7)	7558	−189 (−2.4)	4160	−23 (−0.6)	16 062	−126 (−0.8)
2016	4254	−90 (−2.1)	7994	436 (5.8)	3827	−333 (−8.7)	16 071	9 (0.1)
2017	4073	−181 (−4.3)	7861	−133 (−1.7)	3888	61 (1.6)	15 821	−250 (−1.6)
2018	3604	−469 (−11.5)	7537	−324 (−4.1)	3766	−122 (−3.2)	14 900	−921 (−5.8)

Abbreviations: IMG, international medical graduates; NA, not applicable.

Between 2009 and 2014, the total number of applicants has remained relatively constant, ranging from 14 839 to 16 188. The number of applicants from Muslim-majority nations showed annual increases in most years between 2009 and 2015, with decreases of 2.1% in 2016 (from 4344 to 4254), 4.3% in 2017 (to 4073) and 11.5% in 2018 (to 3604) (Table 3). For applicants from outside the US who were not citizens of Muslim-majority nations, there was an increase in the number of applications in 2016 of 5.8% (from 7558 to 7994), followed by comparatively lower decreases of 1.7% in 2017 (to 7861) and 4.1% in 2018 (to 7537). The group of IMGs with US citizenship also showed a decrease in 2015 (0.6%; from 4183 to 4160) and 2016 (8.7%; to 3827) but an increase of 1.6% (to 3888) in 2017 followed by a decrease of 3.2% (to 3766) in 2018. Between 2016 and 2018, the number of applicants from Muslim-majority countries decreased by 650 (15.3%). For all other IMG groups, the decrease was 519 applicants (4.4%).

Since 2009, most IMG applicants from Muslim-majority countries were citizens of Pakistan (9066 of 38 876 [23.3%]), Egypt (4181 [10.8%]), Nigeria (4167 [10.7%]), Saudi Arabia (2767 [7.1%]), and Iran (2629 [6.8%]). Application trends for these 5 countries are presented in Table 4.

**Table 4.** Educational Commission for Foreign Medical Graduates Applicants from Top 5 Countries of Citizenship at Entry to Medical School

Year	Pakistan	Change (%)	Nigeria	Change (%)	Egypt	Change (%)	Saudi Arabia	Change (%)	Iran	Change (%)
2009	713	NA	331	NA	338	NA	151	NA	250	NA
2010	769	56 (7.9)	391	60 (18.1)	386	48 (14.2)	266	115 (76.2)	272	22 (8.8)
2011	757	−12 (−1.6)	454	63 (16.1)	349	−37 (−9.6)	343	77 (28.9)	288	16 (5.9)
2012	813	56 (7.4)	389	−65 (−14.3)	394	45 (12.9)	350	7 (2.0)	284	−4 (−1.4)
2013	883	70 (8.6)	401	12 (3.1)	485	91 (23.1)	351	1 (0.3)	316	32 (11.3)
2014	1043	160 (18.1)	357	−44 (−11.0)	529	44 (9.1)	303	−48 (−13.7)	301	−15 (−4.7)
2015	1042	−1 (−0.1)	426	69 (19.3)	493	−36 (−6.8)	337	34 (11.2)	281	−20 (−6.6)
2016	1115	73 (7.0)	486	60 (14.1)	479	−14 (−2.8)	311	−26 (−7.7)	265	−16 (−5.7)
2017	1013	−102 (−9.1)	466	−20 (−4.1)	419	−60 (−12.5)	192	−119 (−38.3)	190	−75 (−28.3)
2018	919	−94 (−9.3)	466	0	309	−110 (−26.3)	163	−29 (−15.1)	182	−8 (−4.2)

Abbreviation: NA, not applicable.

## Discussion

The purpose of this study was to describe the current composition of the IMG physician workforce in the US, highlighting the contribution of citizens from Muslim-majority nations. Our analyses indicated that 4.5% of the current active US physician workforce were citizens of a Muslim-majority nation. Some of these IMGs graduated from medical school in a non–Muslim-majority nation but, based on citizenship, could still encounter immigration and visa challenges depending on where they wish to obtain postgraduate education. More than half of all IMGs from Muslim-majority countries were citizens of Egypt, Iran, or Pakistan.

A second aim was to provide longitudinal trends in the number of citizens from Muslim-majority nations seeking ECFMG certification, likely with the intent of securing a GME position in the US. Since 2015, the total number of applicants has decreased for all IMG cohorts but more so for citizens of Muslim-majority nations.

From a migration perspective, it is important to look at the trends in relation to factors that influence immigration in general and physician migration in particular. These are generally explained as a mix of push and pull factors^30^ and include macroeconomic issues, such as labor market demand; political issues, such as security and stability; personal considerations, such as quality of life; and professional conditions, such as advanced training opportunities and wages.^31,32^ However, in the case of physician migration to the US, there are other factors that act as barriers and might affect the decision to seek specialty training in the US. These include, among others, the application process for ECFMG certification, the competition for residency positions, and state rules governing the medical licensure of IMGs.

As a leading indicator, changes in the number and characteristics of medical students and graduates applying for ECFMG certification provides some signal of the influence of various barriers that serve to restrict the flow of IMGs to the US workforce. While our data showed a general increase in applicant numbers between 2009 and 2015, there has been a decrease since 2015. Given that IMGs tend to fill gaps in the physician workforce, this decrease—or even a change in the characteristics of the IMG residency pipeline—could certainly have some future consequences for the US workforce.

There are a number of factors that can help explain why IMGs choose to, or choose not to, attempt ECFMG certification and then compete for GME positions. First, changes to ECFMG certification requirements might act as a deterrent for prospective applicants. The requirements have undergone significant changes during the past 20 years, including the introduction of the ECFMG Clinical Skills Assessment in 1998 and the USMLE Step 2 Clinical Skills in 2004, both of which required candidates to travel to the US to take the examinations. The fees might also be considered prohibitive, especially for IMGs from low-income countries. To be ECFMG certified, IMGs must pass USMLE Step 1, Step 2 Clinical Knowledge, and Step 2 Clinical Skills. For IMGs, the Step 2 Clinical Skills examination alone currently costs $1580; this does not include the costs associated with travel and lodging.^33^

Second, a perceived low likelihood of obtaining a residency position might dissuade potential ECFMG applicants. While this seems plausible, there is little evidence to support it. Since 2011, the National Resident Matching Program has reported annual increases in the number of non–US citizen IMGs who matched.^34^ The 2019 match had the highest match rate in more than 25 years.^35^ However, this might point to a certain self-selection effect: while the number of individuals who submitted final rank order lists has decreased in the last few years, the ratio of applicants successfully obtaining a residency position has increased. Our investigation did not address the relative strength of residency program applicants (ie, their USMLE scores). Therefore, it is unknown whether the IMGs from Muslim-majority countries in our study cohort are of higher (or lower) ability than other IMGs, potentially affecting their chances of securing a residency position. Finally, the establishment of new medical schools in the US and the increasing size of matriculating classes will lead to greater numbers of medical graduates from the US in the future. Coupled with little additional funding for GME positions, obtaining residency positions might become even more competitive for IMGs in the future.

The factors underlying the decrease in ECFMG applications from citizens of Muslim-majority nations are difficult to disentangle. One hypothesis is that the attractiveness of the US as a destination for medical training has waned compared with other countries, such as Australia, Canada, New Zealand, and the UK.^36^ Even the perception of a Muslim ban in the US, whether the legislation is upheld in the courts, could dissuade some medical students and graduates from attempting the ECFMG certification process. Another possibility is that IMGs do not need to leave their home country for specialty training because standards of GME worldwide have been raised and new residency programs have been developed.^37,38,39,40^ Finally, current societal attitudes discourage IMGs from Muslim-majority nations from relocating, often with spouses and other family members,^41,42^ given that their perception of the integration process is negative.

### Limitations

Our study has several limitations. First, the AMA Masterfile has been criticized for overcounting or undercounting certain subcategories.^43^ It remains the best available data source for enumerating the US physician workforce^44,45^ and has been used it in previous investigations.^46,47,48^ Second, while we looked at longitudinal data, we did not track the movement of individual physicians. This means that numbers are reported at an aggregate level, and remigration to country of origin has not been captured in our data set. Similarly, IMGs from Muslim-majority countries might have undergone a naturalization process and thus obtained US citizenship. In our data set, they would be considered part of the cohort from Muslim-majority countries based on their citizenship at medical school. This could result in an overestimate of the number of practicing IMGs from Muslim-majority countries but would not have affected the applicants for certification because that process takes place before entering the US. Our inclusion of individuals coming from Muslim-majority nations did not consider their individual circumstances or religious affiliation. Additionally, we did not consider individuals from other countries with large Muslim populations, such as India and Ethiopia, because we were primarily interested in country-level trends. Finally, while we described trends in physician migration and ECFMG certification applications, the causes of these trends are not known. Although there was a much larger decrease in ECFMG applications from citizens of Muslim-majority countries between 2017 and 2018, we can only infer that the travel ban was a precipitating factor.

## Conclusions

International medical graduates, including those who were citizens of Muslim-majority nations at entry to medical school, make up a large part of the US physician workforce. Based on the results of this study, the IMG pool is changing, with relatively fewer citizens from Muslim-majority countries attempting examinations required for ECFMG certification.
